# Diffusion weighted imaging changes in extra-abdominal desmoid tumor after cryotherapy

**DOI:** 10.1080/07853890.2023.2174589

**Published:** 2023-02-01

**Authors:** Farah Cadour, Farouk Tradi, Axel Bartoli, Florence Duffaud, Jean-Yves Gaubert

**Affiliations:** a Department of Radiology, La Timone Hospital, Marseille, France; b Department of Oncology, La Timone Hospital, Marseille, France

**Keywords:** Cryotherapy, desmoid tumor, diffusion weighted imaging, magnetic resonance imaging, cryoablation

## Abstract

Desmoid tumors (DT) are rare benign tumors with a local invasion potential and recurrence. It is characterized on histology by an abnormal fibroblastic proliferation in a collagenous stroma, in variable proportions leading to heterogeneity of the lesion signal on magnetic resonance imaging (MRI). Current guidelines propose watchful waiting but in case of progression or symptoms, cryotherapy may be a therapeutic option in its extra-abdominal form. Tumor recurrence is mostly detected based on post-contrast magnetic resonance imaging (MRI). Although DWI sequence is the key-sequence for tumor detection in oncologic imaging, there are very few data in literature on diffusion weighted imaging (DWI) in DT generally and even fewer on DT after cryotherapy. DWI changes after cryotherapy may be confusing and suspicious of residual tumor or tumor recurrence when displaying low ADC values; thus knowledge of possible DWI patterns after cryotherapy of DT seem paramount. We found that the early changes of DT after cryotherapy are hyperintensity on DWI sequence with low ADC values (<1.00 × 10^−3^mm^2^/s), without corresponding enhancement and a later decrease in signal of the treated lesion on DWI. The freezing-thawing cycles of cryotherapy turn DT into gelatinous necrosis with a slow resorption rate, as reported in the only few studies referring of changes of DWI signals after cryotherapy, which are on renal and prostate models. Hyperintensity on DWI with low ADC values may be seen in early MRI follow-up after cryotherapy of extra-abdominal DT, corresponding with tumor necrosis changes and should not be mistaken with recurrence.

**KEY MESSAGES**
- Magnetic resonance imaging is the modality of choice for desmoid tumor (DT) follow-up, mainly based on contrast uptake which make data on diffusion weighted imaging (DWI) very rare.
- Cryotherapy is an accepted therapeutic option for DT that will lead to tumor necrosis.
- Hyperintensity on DWI with low apparent diffusion coefficient values is a possible expected early pattern on DWI after cryotherapy of DT.

## Introduction

Desmoid tumor (DT) are benign locally aggressive neoplasm that typically occurs in young adults, mostly in female, in abdominal or extra-abdominal locations [1]. It is histologically characterized by monoclonal fibroblastic proliferation with a tendency to local recurrence although possible spontaneous regression [2]. Current guidelines recommend intervention on DT only in case of progression, morbidity or symptoms. Due to its rarity [3], there are to date no clear consensus on the best therapeutic management of these tumors. Historically, surgery was the standard treatment however recent guidelines consider more local ablative treatment as an alternative to medical therapies [4,5], which may have a delayed time to action [6]. In terms of local treatment, radiotherapy may offer good control but at the cost of long-term radiotoxicity [7]. In this perspective, cryotherapy has emerged as a safe and effective treatment for extra-abdominal DT [8–12]. In reported studies, recurrence has essentially been evaluated through magnetic resonance imaging (MRI) [13,14], however to the best of our knowledge, no study were specifically designed to discuss the signal evolution of DT on MRI after cryotherapy. Additionally, most reported studies referred to contrast product uptake for recurrence detection [15,16]. DT on MRI may display homogeneous, heterogeneous, or no significant enhancement [17]. This variability in signal reflects differences in the composition and cellularity. Knowing the sensitivity of diffusion weighted imaging (DWI) in cellularity detection and its broad use in this goal in oncology imaging, studies investigating the place of DWI in DT are surprisingly rare [18–20]. Thus, it is even rarer to find data on DWI signal evolution after DT treatment by cryotherapy.

## Discussion

### The place of DWI in MRI for DT

Histologically, DT are composed of dense collagenous stroma and uniform fibroblasts and are usually positive for SMA and β_1_-catenin [21]. MRI is the modality of choice for DT because of its excellent contrast resolution for soft-tissue imaging. DT present on MRI mainly a fascicular configuration, both in its primitive and recurrent form [13,14]. If not infiltrative, extra-abdominal DT is usually a large well-defined mass, which can present the tail sign corresponding to its extension along the fascia [22]. Its signal is heterogeneous and differs depending on its cellularity and fibrous content [17], with variable T2 signal and usually low/iso T1 signal. Increased T2 signal will correlate with high content of spindle cells whereas low T2 signal will correlate to hypocellularity and dense collagen [23]. Enhancement is, in almost 90% of the DT, moderate to marked, especially in case of hypercellular DT [24]. DWI is the archetype of the sequence when looking for cellularity, even more in the field of oncologic imaging. Surprisingly, DWI seems to lose its place of choice when talking about DT. Even in the largest prospective European cohort to date [11], there is no place for DWI in the MRI protocol. We can wonder whether DWI is relevant for DT. Some studies, remaining however rare, have shown that DWI may help to differentiate DT with malignant tumors [18,25], with a mean ADC of DT of 1.36 ± 0.48 × 10^–^³mm^2^/s *vs.* 0.88 ± 0.20 × 10^–^³mm^2^/s for malignant soft tissue tumors. DWI seems also to correlate with content of the DT, with higher ADC values in DT in case of low cellularity and fibrous content [18,25]. The remaining question is the place of DWI in the follow-up of patients after DT treatment, treatment that gives a growing place to cryotherapy, and thus the place of DWI after cryotherapy.

### DWI after cryotherapy

Current guidelines [26] recommend watchful waiting but in case of progressive or symptomatic disease, individualized strategy must be based on a multidisciplinary basis. In addition to being a key-treatment of soft-tissue lesions (both for malignant and symptomatic benign), cryotherapy is now a minimally invasive recognized therapeutic option for the extra-abdominal location of DT [11].

MRI is the modality of choice for extra-abdominal recurrence, whereas CT is the preferred modality for abdominal one [23]. The traditional follow-up schedule is every three months the first year after the first control one month after the procedure [11,27]. Most studies rely on contrast uptake to look for recurrence, but to our knowledge, none have investigated DWI for recurrence detection and none have reported DT features on DWI after cryotherapy. Interpretation of the changes of DT signal after cryotherapy may be difficult, especially due to the lack of reports in the literature of the possible expected changes after cryotherapy of DT. There are only few studies that reported DWI after cryotherapy, but that were studies focused on renal cell carcinoma in humans or prostate cryotherapy in canine models [28–31]. In renal and prostate models, there were also high signals in DWI with low ADC in the first months after cryotherapy, slowly becoming hypointense at 6–9 month follow-up; and which they have suggested being due to first tissue swelling and later scar formation [28]. In cryotherapy, freezing-thawing cycles cause damage to the cell membrane leading to apoptosis [32] then turning solid tumors like DT into gelatinous necrosis with a slow resorption rate [11].

### A possible DWI pattern post cryotherapy of DT

**Figure 1.**
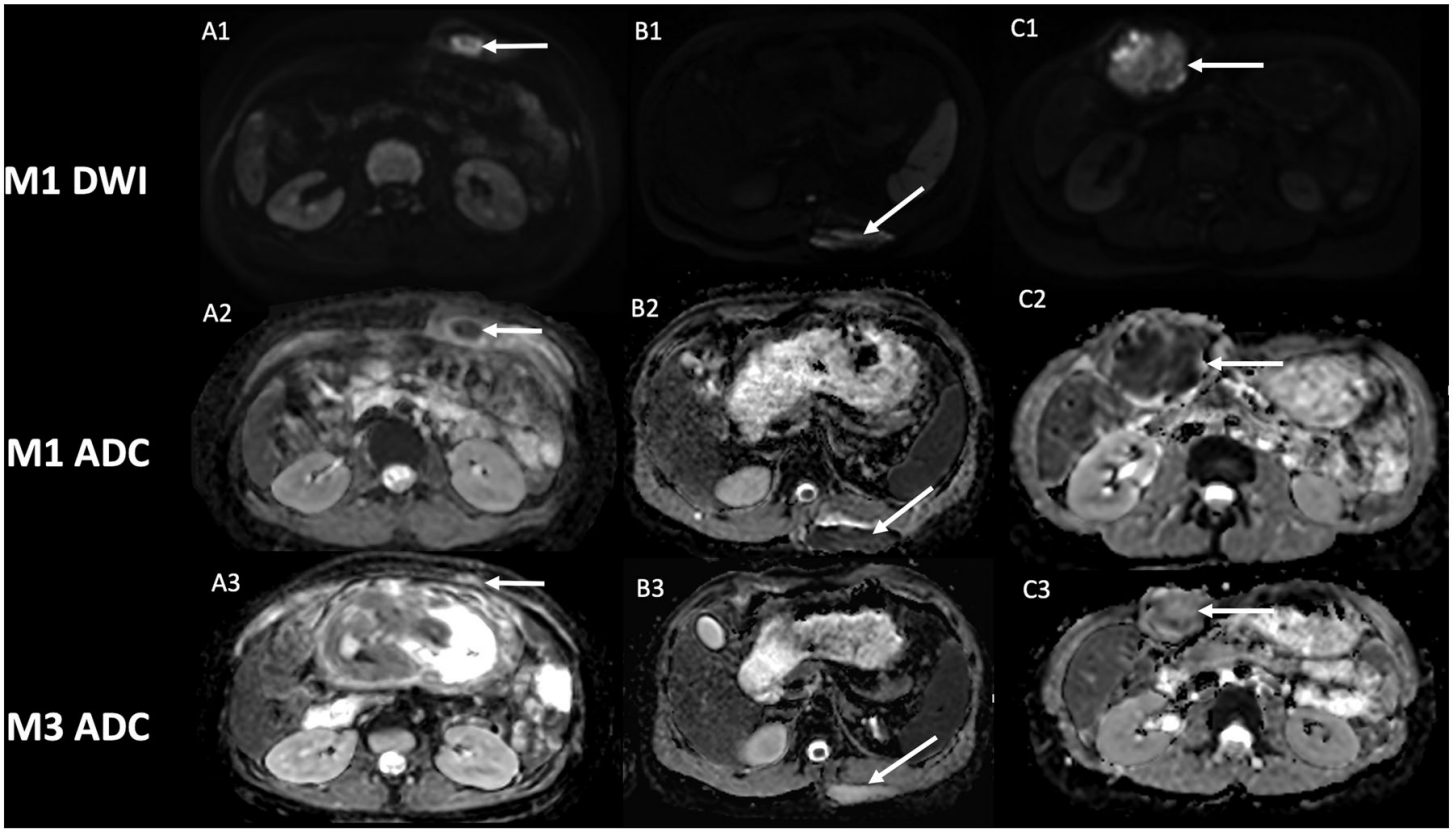
Evolution of diffusion weighted-imaging (DWI) after cryotherapy of extra-abdominal desmoid tumors. Post-cryotherapy imaging of three different patients showing at one-month control a hyperintensity on DWI (A1, B1, C1) with low apparent diffusion coefficient (ADC) within the core of the phantom of the lesion (arrow), and subsequent increase of ADC value at 3-month control (A3, B3, C3). A2: ADC = 0.84 × 10^−3^ mm^2^/s; A3: ADC = 1.82 × 10^−3^ mm^2^/s. B2: ADC = 0.96 × 10^−3^ mm^2^/s; B3: ADC = 2.12 × 10^−3^ mm^2^/s. C2: ADC = 0.90 × 10^−3^ mm^2^/s; C3: ADC = 1.52 × 10^−3^ mm^2^/s.

**Figure 2.**
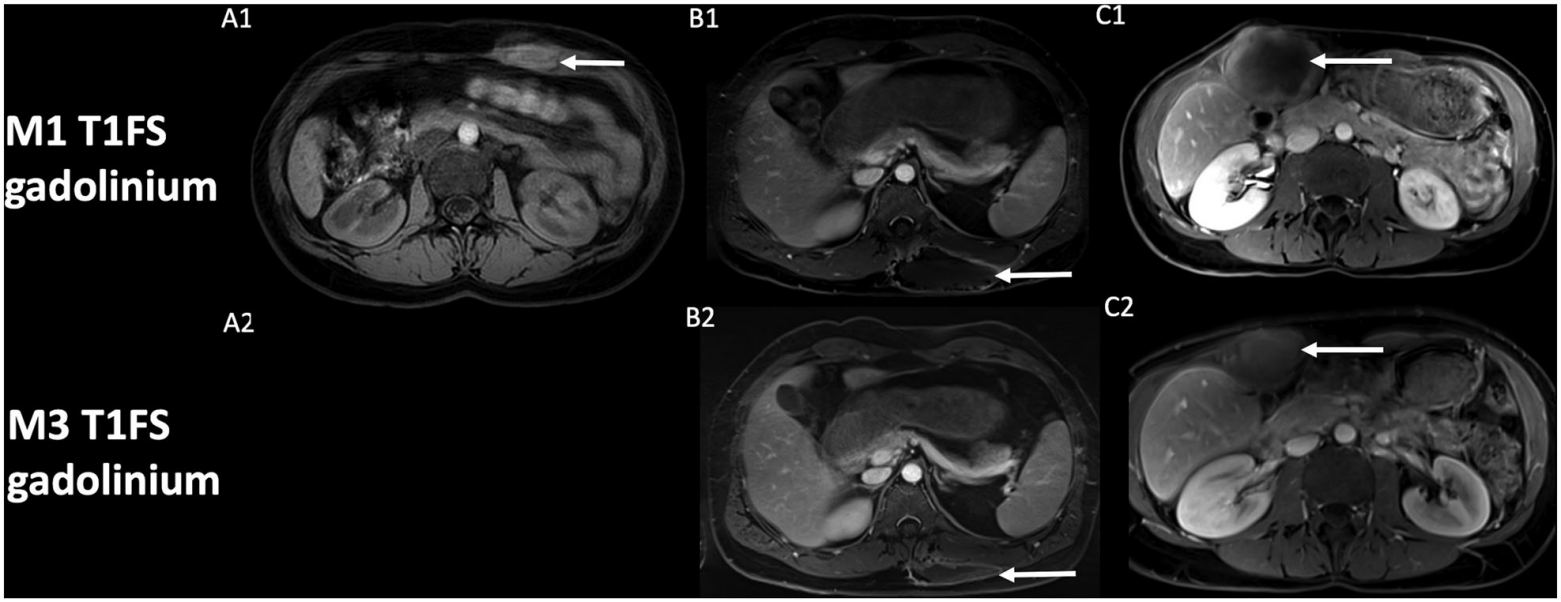
Evolution of contrast uptake after cryotherapy of extra-abdominal desmoid tumors. Post-cryotherapy imaging of three different patients showing at one-month control (A1, B1, C1) the corresponding T1 fat-sat with gadolinium of the core of the phantom of the lesion (arrow), with subsequent decrease in size of the scar lesion at 3-month control (A2, B2, C2). Note that control at 3-month for patient A (A2) was not available at the request of the patient to stop the exam.

The classic MRI protocol in our institution, which is a referral expert center for DT, includes DWI, in addition to pre- and post-contrast imaging. We have identified in several patients with extra-abdominal DT successfully treated by cryotherapy at first control MRI, one month after cryotherapy, low ADC values (mean value 0.90 × 10^−3^ mm^2^/s), in the site of cryotherapy. That made us fear a residual tumor or early recurrence, especially knowing the added-value of DWI in oncology and recurrence detection of malignant lesion [33–35]. The zone of low ADC values corresponded with zone of T1 hyperintensity and no contrast uptake. Analysis in retrospect of the positioning of our cryoprobes broadly encompassing the lesion, and because of the lack of enhancement, we considered these findings as expected early changes of DT after cryotherapy. Moreover, the low ADC values were located in the core of the lesion phantom, which is highly atypical in recurrence that often occurs in tumor margins. Distant follow-up also confirmed the early changes in DWI, with an increase of ADC values, still no contrast uptake, and correlating with a decrease in size of the scar of cryotherapy (Figures 1 and 2).

DWI findings should therefore always be confronted to contrast uptake, which is still the current standard of DT follow-up on MRI after treatment and help to achieve high performances for detecting recurrent DT [14]. DWI findings should also always be confronted to change in the size of a treated lesion: a treated lesion should progressively involute, especially at distance of the treatment. Even if signal changes after cryotherapy may be confusing, a possible DWI pattern in DT treated by cryotherapy is initially low ADC values. These findings could represent simple necrotic and hemorrhagic changes due to liquid tumor necrosis, in the initial stages, and should not be mistaken with recurrence. A critical analysis of cryoprobes positioning and lesion coverage during cryotherapy, the absence of suspicious enhancement and close follow-up by MRI in addition to clinical symptoms, are paramount. Further studies with large cohort of patients and implementation of DWI in the protocol of follow-up of DT after cryotherapy would be of interest to present all the possible DWI findings and to confirm our findings.

## Conclusion

Few data on DWI features of DT are available and even fewer data exist on DWI after cryotherapy. We suggest that low ADC values in early follow-up of DT after cryotherapy are possible and are due to tumor necrosis. These changes should not be mistaken with recurrence, especially if enough volume coverage was performed during cryotherapy and if no enhancement is present. Further larger studies are however needed to support our findings.

## Data Availability

Data sharing is not applicable to this article as no new data were created or analyzed in this study.

## References

[1] Moorthy GD, Amini B, Nikolaidis P, et al. Current update on desmoid fibromatosis. J Comput Assist Tomogr. 2019;43(1):29–38. 30211798 10.1097/RCT.0000000000000790 PMC6331223

[2] Jo VY, Fletcher CDM. WHO classification of soft tissue tumours: an update based on the 2013 (4th) edition. Pathology. 2014;46(2):95–104. 24378391 10.1097/PAT.0000000000000050

[3] Penel N, Coindre JM, Bonvalot S, et al. Management of desmoid tumours: a nationwide survey of labelled reference Centre networks in France. Eur J Cancer. 2016;58:90–96. 26974708 10.1016/j.ejca.2016.02.008

[4] Group DTW. The management of desmoid tumours: a joint global consensus-based guideline approach for adult and paediatric patients. Eur J Cancer. 2020;127:96–107. 32004793 10.1016/j.ejca.2019.11.013

[5] Walczak BE, Rose PS. Desmoid: the role of local therapy in an era of systemic options. Curr Treat Options in Oncol. 2013;14(3):465–473. 10.1007/s11864-013-0235-7 23760919

[6] Gounder MM, Mahoney MR, Van Tine BA, et al. Sorafenib for advanced and refractory desmoid tumors. N Engl J Med. 2018;379(25):2417–2428. 30575484 10.1056/NEJMoa1805052 PMC6447029

[7] Looi WS, Indelicato DJ, Rutenberg MS. The role of radiation therapy for symptomatic desmoid tumors. Curr Treat Options Oncol. 2021;22(4):34. 33649873 10.1007/s11864-021-00831-6

[8] Vora BMK, Munk PL, Somasundaram N, et al. Cryotherapy in extra-abdominal desmoid tumors: a systematic review and meta-analysis. PLoS One. 2021;16(12):e0261657. 34941915 10.1371/journal.pone.0261657 PMC8699690

[9] Havez M, Lippa N, Al-Ammari S, et al. Percutaneous image-guided cryoablation in inoperable extra-abdominal desmoid tumors: a study of tolerability and efficacy. Cardiovasc Intervent Radiol. 2014;37(6):1500–1506. 24402645 10.1007/s00270-013-0830-9

[10] Schmitz JJ, Schmit GD, Atwell TD, et al. Percutaneous cryoablation of extraabdominal desmoid tumors: a 10-year experience. AJR Am J Roentgenol. 2016;207(1):190–195. 27064168 10.2214/AJR.15.14391

[11] Kurtz JE, Buy X, Deschamps F, et al. CRYODESMO-O1: a prospective, open phase II study of cryoablation in desmoid tumour patients progressing after medical treatment. Eur J Cancer. 2021;143:78–87. 33290994 10.1016/j.ejca.2020.10.035

[12] Saltiel S, Bize PE, Goetti P, et al. Cryoablation of extra-abdominal desmoid tumors: a single-center experience with literature review. Diagnostics. 2020;10(8):556. 32759783 10.3390/diagnostics10080556 PMC7460498

[13] Sedaghat S, Surov A, Krohn S, et al. Configuration of primary and recurrent aggressive fibromatosis on contrast-enhanced MRI with an evaluation of potential risk factors for recurrences in MRI follow-up. Rofo MAI. 2020;192(5):448–457. 10.1055/a-1022-4546 31622987

[14] Sedaghat S, Sedaghat M, Krohn S, et al. Long-term diagnostic value of MRI in detecting recurrent aggressive fibromatosis at two multidisciplinary sarcoma centers. Eur J Radiol. 2021;134:109406. 33254066 10.1016/j.ejrad.2020.109406

[15] Kujak JL, Liu PT, Johnson GB, et al. Early experience with percutaneous cryoablation of extra-abdominal desmoid tumors. Skeletal Radiol. 2010;39(2):175–182. 19768644 10.1007/s00256-009-0801-z

[16] Cornelis F, Havez M, Lippa N, et al. Radiologically guided percutaneous cryotherapy for soft tissue tumours: a promising treatment. Diagn Interv Imaging. 2013;94(4):364–370. 23491212 10.1016/j.diii.2013.02.001

[17] Azizi L, Balu M, Belkacem A, et al. MRI features of mesenteric desmoid tumors in familial adenomatous polyposis. AJR Am J Roentgenol. 2005;184(4):1128–1135. 15788583 10.2214/ajr.184.4.01841128

[18] Oka K, Yakushiji T, Sato H, et al. Ability of diffusion-weighted imaging for the differential diagnosis between chronic expanding hematomas and malignant soft tissue tumors. J Magn Reson Imaging. 2008;28(5):1195–1200. 18972359 10.1002/jmri.21512

[19] Einarsdóttir H, Karlsson M, Wejde J, et al. Diffusion-weighted MRI of soft tissue tumours. Eur Radiol. 2004;14(6):959–963. 14767604 10.1007/s00330-004-2237-0

[20] Khanna M, Ramanathan S, Kambal AS, et al. Multi-parametric (mp) MRI for the diagnosis of abdominal wall desmoid tumors. Eur J Radiol. 2017;92:103–110. 28624006 10.1016/j.ejrad.2017.04.010

[21] Rosa F, Martinetti C, Piscopo F, et al. Multimodality imaging features of desmoid tumors: a head-to-toe spectrum. Insights Imag. 2020;11(1):103. 10.1186/s13244-020-00908-0 PMC7520866 32986198

[22] Dinauer PA, Brixey CJ, Moncur JT, et al. Pathologic and MR imaging features of benign fibrous soft-tissue tumors in adults. Radiographics. 2007;27(1):173–187. 17235006 10.1148/rg.271065065

[23] Braschi-Amirfarzan M, Keraliya AR, Krajewski KM, et al. Role of imaging in management of desmoid-type fibromatosis: a primer for radiologists. Radiographics. 2016;36(3):767–782. 27163593 10.1148/rg.2016150153

[24] Robbin MR, Murphey MD, Temple HT, et al. Imaging of musculoskeletal fibromatosis. Radiographics. 2001;21(3):585–600. 11353108 10.1148/radiographics.21.3.g01ma21585

[25] Oka K, Yakushiji T, Sato H, et al. Usefulness of diffusion-weighted imaging for differentiating between desmoid tumors and malignant soft tissue tumors. J Magn Reson Imag. 2011;33(1):189–193. 10.1002/jmri.22406 21182138

[26] Gronchi A, Miah AB, Dei Tos AP, et al. Soft tissue and visceral sarcomas: ESMO-EURACAN-GENTURIS clinical practice guidelines for diagnosis, treatment and follow-up. Ann Oncol. 2021;32(11):1348–1365. 34303806 10.1016/j.annonc.2021.07.006

[27] Maas M, Beets-Tan R, Gaubert JY, et al. Follow-up after radiological intervention in oncology: ECIO-ESOI evidence and consensus-based recommendations for clinical practice. Insights Imag. 2020;11(1):83. 10.1186/s13244-020-00884-5 PMC7366866 32676924

[28] Lee HJ, Chung HJ, Wang HK, et al. Evolutionary magnetic resonance appearance of renal cell carcinoma after percutaneous cryoablation. Br J Radiol. 2016;89(1065):20160151. 27401340 10.1259/bjr.20160151 PMC5124922

[29] Butts K, Daniel BL, Chen L, et al. Diffusion-weighted MRI after cryosurgery of the canine prostate. J Magn Reson Imag. 2003;17(1):131–135. 10.1002/jmri.10227 12500282

[30] Chen J, Daniel BL, Diederich CJ, et al. Monitoring prostate thermal therapy with diffusion-weighted MRI. Magn Reson Med. 2008;59(6):1365–1372. 18506801 10.1002/mrm.21589

[31] Lopes Dias J, Lucas R, Magalhães Pina J, et al. Post-treated prostate cancer: normal findings and signs of local relapse on multiparametric magnetic resonance imaging. Abdom Imag. 2015;40(7):2814–2838. 10.1007/s00261-015-0473-1 26105522

[32] Efrima B, Ovadia J, Drukman I, et al. Cryo-surgery for symptomatic extra-abdominal desmoids. A proof of concept study. J Surg Oncol. 2021;124(4):627–634. 34043245 10.1002/jso.26528

[33] Del Grande F, Subhawong T, Weber K, et al. Detection of soft-tissue sarcoma recurrence: added value of functional MR imaging techniques at 3.0 T. Radiology. 2014;271(2):499–511. 24495264 10.1148/radiol.13130844

[34] ElDaly MM, Moustafa AFI, Abdel Meguid Sms Shokry AM, et al. Can MRI diffusion-weighted imaging identify postoperative residual/recurrent soft-tissue sarcomas? Indian J Radiol Imag. 2018;28(1):70–77. 10.4103/ijri.IJRI_251_17 PMC5894324 29692531

[35] Messina C, Bignone R, Bruno A, et al. Diffusion-weighted imaging in oncology: an update. Cancers. 2020;12(6):1493. 32521645 10.3390/cancers12061493 PMC7352852

